# Direct and transgenerational effects of low doses of perinatal di-(2-ethylhexyl) phthalate (DEHP) on social behaviors in mice

**DOI:** 10.1371/journal.pone.0171977

**Published:** 2017-02-15

**Authors:** Kayla M. Quinnies, Erin P. Harris, Rodney W. Snyder, Susan S. Sumner, Emilie F. Rissman

**Affiliations:** 1 Department of Biochemistry and Molecular Genetics and Neuroscience Graduate Program, University of Virginia School of Medicine, Charlottesville, VA, United States of America; 2 Discovery Science Technology, RTI International, Research Triangle Park, NC, United States of America; 3 Center for Human Health and the Environment, North Carolina State University, Raleigh, NC, United States of America; University of Missouri Columbia, UNITED STATES

## Abstract

Di-(2-ethylhexyl) phthalate (DEHP) is an endocrine disrupting chemical commonly used as a plasticizer in medical equipment, food packaging, flooring, and children’s toys. DEHP exposure during early development has been associated with adverse neurobehavioral outcomes in children. In animal models, early exposure to DEHP results in abnormal development of the reproductive system as well as altered behavior and neurodevelopment. Based on these data, we hypothesized that developmental exposure to DEHP would decrease social interactions and increase anxiety-like behaviors in mice in a dose-dependent manner, and that the effects would persist over generations. C57BL/6J mice consumed one of three DEHP doses (0, 5, 40, and 400 μg/kg body weight) throughout pregnancy and during the first ten days of lactation. The two higher doses yielded detectable levels of DEHP metabolites in serum. Pairs of mice from control, low, and high DEHP doses were bred to create three dose lineages in the third generation (F3). Average anogenital index (AGI: anogenital distance/body weight) was decreased in F1 males exposed to the low dose of DEHP and in F1 females exposed to the highest dose. In F1 mice, juvenile pairs from the two highest DEHP dose groups displayed fewer socially investigative behaviors and more exploratory behaviors as compared with control mice. The effect of DEHP on these behaviors was reversed in F3 mice as compared with F1 mice. F1 mice exposed to low and medium DEHP doses spent more time in the closed arms of the elevated plus maze than controls, indicating increased anxiety-like behavior. The generation-dependent effects on behavior and AGI suggest complex mechanisms by which DEHP directly impacts reproductive and neurobehavioral development and influences germline-inherited traits.

## Introduction

Di-(2-ethylhexyl) phthalate (DEHP) is a synthesized component of flexible plastics used to make polyvinyl chloride (PVC), some types of packaging, medical tubing, synthetic flooring and other products [1–3]. Concentrations of DEHP in manufactured materials may reach 40% by weight and over 98% of the US population has detectable levels of its metabolites in urine [4]. This endocrine disrupting chemical (EDC) crosses the placenta [5], is absorbed through the skin [6], and its metabolites are found in human breast milk [7]. Daily intake in humans has been estimated between 6–21 μg/kg, with children at the higher end of this range [8]. Over 85% of the studies included in a recent review reported levels of phthalate exposure in children above the maximum reference dose set by the United States Environmental Protection Agency [9].

DEHP disrupts endocrine function in various glands and tissues throughout the body, but is most commonly considered to be anti-androgenic [10]. Maternal levels of the major DEHP metabolite, mono-(2-ethylhexyl) phthalate (MEHP), are correlated with decreased levels of steroid hormones in human male infants [11], and decreased function of Sertoli and Leydig cells in adult rodents [12, 13]. DEHP has non-monotonic dose-response effects. For example, in mice, high doses cause a drop in maternal and fetal serum testosterone, whereas lower levels of DEHP increase testosterone [14]. Anogenital distance (AGD; distance from anus to genitalia) is determined by androgen action during early development: males have longer AGD than females, and this can be perturbed by manipulations of androgen levels. Increased gestational DEHP levels correlate with decreased AGD in male rodents [14–16] and humans [17–19], which further indicates its anti-androgenic actions.

Human epidemiological studies have also revealed associations between prenatal phthalate exposure and adverse neurodevelopmental outcomes in children [20–26]. DEHP metabolite levels in utero are associated with decreased masculine play in boys [27]. Boys with attention deficit hyperactivity disorder (ADHD) have higher urinary concentrations of DEHP metabolites, which are negatively correlated with cortical thickness [28]. Phthalates have also been implicated in the pathogenesis of autism spectrum disorder (ASD) [29]. Children with autism spectrum disorder (ASD) have higher urinary levels of DEHP metabolites [30, 31] and show impaired glucuronidation of DEHP metabolites as compared with typically developing children [32]. It is worth noting that DEHP levels are positively correlated with fast food consumption [4]. Thus studies that show correlations between behaviors and current levels of DEHP in children may reflect food choices.

In animals, DEHP exposure during several developmental periods (gestation and/or suckling, puberty, or adulthood) increases anxiety and depression-like behavior [12, 33, 34]. DEHP exposure during puberty decreases adult social interactions in female mice, but enhances interactions in males [33, 35]. We recently reported sex-specific effects of DEHP three generations removed from the initial gestational exposure (F3); juvenile male F3 mice from a DEHP lineage displayed more digging and less grooming than controls in social interaction tests [36]. While the doses used in this study were relatively high (150 and 200 mg/kg body weight), these results were the first evidence of a transgenerational effect of DEHP exposure on behavior.

In the present report, we evaluated three low doses of DEHP: 5, 40, and 400 μg/kg, all of which are below the no observed effect level (NOAEL, 4.8 mg/kg/day) for DEHP and comparable to normal human intake [37]. We detected significant and complex transgenerational effects of DEHP exposure on AGI, social interactions, as well as a generation-specific effect on anxiety-like behavior. This is first report of social behavior changes in response to perinatal low dose DEHP exposure, and the first time that transgenerational effects have been reported in a low dose exposure lineage.

## Materials and methods

### Animals

Male and female C57BL/6J mice originally purchased from Jackson Laboratory (Bar Harbor, ME) were used in our colony at the University of Virginia. All procedures followed were approved by the University of Virginia Animal Care and Use Committee guidelines. All animals were maintained on a 12:12 light/dark cycle (lights on at 1300h), provided with a diet low in phytoestrogens (Harlan Laboratories, Indianapolis, IN #2918), and water *ad libitum*. Females were paired with males shortly before lights off each day and were checked for mating plugs the next day. Dams were assigned to a dose group at random as they became pregnant, with the goal of keeping groups as equal in size as possible. Males were assigned at random from a large group of breeding males. These pairs were the original generation (F0). Pairs were separated and females were individually housed on the day the mating plug was observed. On each day of gestation and continuing until the litter was 10 days of age, dams consumed a Cocoa Puff coated in 50 μL of stripped corn oil containing doses of DEHP equivalent to 0, 5 (“Low dose”), 40 (“Medium dose”), or 400 (“High dose”) μg/kg body weight per day. Doses were presented before lights off and females were observed to ensure they consumed the entire treat. On postnatal day 1 (P1), we recorded sex, body weight, and AGD for each pup in all litters. Following these measurements, all litters were culled to 6 pups with a sex ratio as close as possible to equal; no single sex litters were used. Pups selected for culling were randomly chosen within each sex and not based on bodyweight or AGD measurements. Culled pups were sacrificed by first reducing their body temperatures and then using cervical dislocation. Mice were weaned on P21, housed in same-sex, same-litter groups and randomly assigned to behavioral testing or breeding. The mice remained housed in groups during behavioral testing. Mice were only tested on one task using no more than two mice from the same litter, one of each sex. F1 pairs were made with dose-matched non-siblings to create F2 offspring. The same breeding protocol was followed with the F2 mice to create F3 mice. Due to space and cost considerations we conducted the transgenerational work with lineages produced from the control (0 dose), low (5 μg/kg), and highest dose (400 μg/kg) of DEHP. Observers blind to the treatments of the mice scored all behaviors.

### MEHP analysis

A cohort of females was paired, checked daily for plugs, and given a daily dose of DEHP or vehicle as previously described. The day we noted the plug was embryonic day 0.5 (E0.5). These dams were sacrificed at E18.5, no more than three hours after consumption of the daily DEHP dose. After anesthesia with isoflurane, animals were euthanized by decapitation and trunk blood was collected from the dams and embryos (pooled by litter). We collected serum from 4 control dams, 2 control embryo pools, 6 low dose dams, 1 low dose embryo pool, 3 medium dose dams, 1 medium dose embryo pool, 6 high dose dams, and 3 high dose embryo pools. Serum was frozen on dry ice, stored at -80°C, and later assayed for DEHP metabolites using a method modified from [38] as described below.

Standards were purchased from Toronto Research Chemicals (Toronto, Ontario, Canada) and standard curve spiking solutions were prepared in methanol. The standard curve for LC/MS/MS analysis consisted of concentrations ranging from 1 to 1000 ng/mL. Internal standard (MEHP-d_4_) was added at 5 μL per sample from a solution in methanol at a concentration of 1 μg/mL. Serum (50 μL) was aliquoted into a polypropylene microfuge tube, and 150 μL methanol was added as well as internal standard. Samples were vortexed, centrifuged (13000 rpm for 10 minutes) and 50 μL of the supernatant was transferred to a glass Agilent LCMS low volume insert and mixed with 50 μL of 5 mM ammonium acetate. For standards, 50 μL of blank mouse serum was mixed with 5 μL of spiking solution containing each compound and extracted as per standards. Standard curves were run prior to mouse serum samples and after mouse serum samples. Quality control samples were included in the middle of the analysis. Standards were within 15% of nominal or 20% of LOQ. Samples were analyzed using a Waters Acquity UPLC (Milford, MA, USA) coupled to an Applied Biosystems/Sciex API 5000 Mass Spectrometer (Concord, Ontario, Canada) with an electrospray ion source.

### Body weight and AGD

Prior to culling litters on P1, we recorded sex, body weight, and AGD for each pup in all litters. For the F1 litters, we recorded measurements from 6 control litters (N = 18 males, N = 17 females), 11 low dose litters (N = 34 males, N = 28 females), 10 medium dose litters (N = 28 males, N = 37 females), and 7 high dose litters (N = 17 males, N = 15 females). For the F3 generation we took measurements from 9 control litters (N = 19 males, N = 23 females), 7 low dose lineage litters (N = 28 males, N = 15 females), and 7 high dose lineage litters, (N = 16 males, N = 22 females).

### Maternal behaviors

We observed a total of 34 F0 dams: Control N = 10, Low dose N = 7, Medium dose N = 9, High dose N = 8. We also observed 16 F2 dams with their F3 litters: Control N = 5, Low dose N = 5, High dose N = 6. Both F0 and F2 dams were observed in their home cages with their litters for 30 minutes during the dark (under red lights) portion of the light:dark cycle. Observations were made on postnatal days 2, 4, and 6. Scan-sampling methods were used to record maternal behaviors: every 15 seconds we noted if the dam was on or off the nest and recorded her behaviors. Behaviors on the nest were: licking and grooming pups, self-grooming, nursing, nest building and hovering. Behaviors off the nest were: eating/drinking, self-grooming, and digging/climbing [39].

### Social interaction test

Age-, dose- and sex-matched pairs of non-sibling juvenile mice (between P28-32) were individually habituated to novel cages containing only clean bedding for 10 minutes, and recorded together in another clean cage for 30 minutes. Tests were conducted during the dark portion of the light cycle under red lights. Behaviors were scored from videos. Scan-sampling was used: the behavior of each mouse was observed and recorded every 15 seconds. Individuals in the pair were identified by a Sharpie black stripe on the tail of one of the mice. Each video was scored twice and the total number of observations was based on individuals in the pair. Interactive behaviors recorded included: side-by-side sitting, social grooming, investigation (sniffing), following, crawling, approaching, and circling the partner. Independent behaviors were: exploring the cage, self-grooming alone, sitting alone, and jumping. In addition, we calculated total interactive and total independent behaviors by summing the frequencies of each event.

### Elevated plus maze

Juvenile (P30-35) F1 and F3 mice were habituated to the behavioral testing room for at least 30 minutes during the dark cycle under red lights, then placed in the center of the elevated plus maze (Columbus Instruments; wall height: 6“, arm length: 11.75”, arm width: 2”) and recorded for 5 minutes. The total time spent in the closed and open arms and the numbers of crosses through the center were scored from the video recordings using Noldus Observer.

### Statistical analysis

All analyses were done using NCSS 2007. Interactions between juvenile pairs and dams with pups were expressed as a proportion of total activity; we used an arcsine transformation to normalize these data. We did not continue to breed the mice produced from the medium dose (40 μg/kg) to the third generation, therefore we had 4 dose groups in F0 dams and F1 offspring, and 3 doses in F2 dams and their F3 offspring. The generations were analyzed separately with two-way ANOVAs (sex and dose). To compare the generations, we used three-way ANOVAs (sex, dose and generation), only including the 3 doses common to both F1 and F3 (Control, low dose, and high dose groups). Maternal behaviors were analyzed with repeated measures ANOVAs with pup age as the within subjects factor. All ANOVAs were followed by Bonferroni-tests corrected multiple comparisons to evaluate pairwise interactions. F statistics and degrees of freedom for all analyses can be found in S1 and S2 Tables.

## Results

### Low concentrations of DEHP metabolites in serum

The highest dose, 400 μg/kg, yielded detectable MEHP and 5-OH-MEHP levels in all serum samples measured. Serum concentrations of MEHP for 400 μg/kg dosed dams averaged 160 ± 17.2 ng/ml and 1.6 ± 0.4 ng/mL for 5-OH-MEHP. Metabolite concentrations of pooled embryo serum from 400 μg/kg dosed dams averaged 96.7 ± 36.9 ng/ml for MEHP and 1 ± 0.20 ng/ml for 5-OH-MEHP. These values are within the range of levels measured in human blood [40]. Two of the three serum samples from dams dosed at 40 μg/kg had detectable MEHP levels (mean ± SEM: 14.2 ± 12.4 ng/ml) and one had detectable 5-OH-MEHP levels (0.54 ng/ml). Metabolite levels in pooled serum from embryos of 40 μg/kg-dosed dams were undetectable. DEHP and metabolite levels in control and low dose pregnant females and their E18.5 embryos were below the detection limit (LOD) of the assay (LOD for MEHP and 5-OH-MEHP were estimated at 0.1 ng/mL).

### Effects of DEHP on AGI and body weights

DEHP exposure decreased AGI in males and females in the F1 generation and in F3 female mice from the lowest dose lineage had larger AGI than the controls. We evaluated males and females separately because of the substantial sex difference in AGI measurements. In F1 males, there was a significant dose effect; AGIs of low dose males were smaller than controls (p<0.05, Table 1). In F1 females, the high dose (400 μg/kg) group had smaller AGIs than both the control and low dose groups (p<0.01). Body weights on P1 were not affected by DEHP in either sex (p>0.05).

**Table 1:** Effects on AGI and body weights in F1 and F3 generations.

Generation	Males	AGI (mm/g)	Body Weight (g)	Females	AGI (mm/g)	Body Weight (g)
**F1**	**Control (18)**	1.17 ± 0.05	1.34 ± 0.05	**Control (17)**	0.73 ± 0.03	1.30 ± 0.03
	**5 μg/kg (34)**	1.03 ± 0.02^**^	1.37 ± 0.02	**5 μg/kg (28)**	0.72 ± 0.02	1.31 ± 0.02
	**40 μg/kg (28)**	1.03 ± 0.03	1.38 ± 0.02	**40 μg/kg (37)**	0.65 ± 0.03	1.34 ± 0.02
	**400 μg/kg (17)**	1.07 ± 0.03	1.31 ± 0.03	**400 μg/kg (15)**	0.61 ± 0.05*	1.37 ± 0.03
**F3**	**Control (19)**	1.07 ± 0.03	1.31 ± 0.03	**Control (23)**	0.63 ± 0.02	1.32 ± 0.02
	**5 μg/kg (28)**	1.08 ± 0.02	1.29 ± 0.02	**5 μg/kg (15)**	0.71 ± 0.02***	1.19 ± 0.03***
	**400 μg/kg (16)**	1.02 ± 0.03	1.35 ± 0.03	**400 μg/kg (22)**	0.64 ± 0.009	1.30 ± 0.02

Mean ± SEM. Number of pups per group in (). Data were collected on postnatal day 1.

*Significantly different from same-sex F1 control and 5 μg/kg groups, p<0.05.

**Significantly different from same-sex F1 control group, p<0.05.

***Significantly different from all other same-sex F3 groups, p<0.05.

Number of F1 litters per group: Control N = 6 litters, 5 μg/kg N = 10 litters, 40 μg/kg N = 10 litters, 400 μg/kg N = 6 litters. Number of F3 litters per group: Control lineage N = 9 litters, 5 μg/kg lineage N = 7 litters, 400 μg/kg lineage N = 6 litters.

In the F3 generation, no differences in AGI were observed in males (p<0.05) and there were no effects of DEHP lineage on body weights in F3 males (p>0.05). Females in the low dose lineage had larger AGIs than both controls and high dose lineage females (p<0.01). In addition, we found an effect of DEHP dose on body weight in females (p<0.001, Table 1) such that low dose lineage females weighed less than both other groups. It is likely that these body weight differences produced the effects on AGI. Comparison of F1 and F3 generations revealed transgenerational effects in females. Dose produced an effect on AGI (p<0.001); high dose females had smaller AGIs compared with the control and low dose females. We also found a dose by generation interaction (p<0.05, S1 Table). Additionally, we found effects of generation (p<0.01), dose, and an interaction for female body weights (p<0.05, S1 Table). F1 females were heavier than F3 females, and the controls and highest dose groups were heavier than the low dose group. The interactions reveal that the low dose F3 mice were lighter than all other groups, except the F1 controls. In males, body weights and AGI were not affected by DEHP dose or generation (p>0.05, S1 Table).

### Pup age and generation affects maternal behaviors

The original (F0) dams did not display any DEHP-related differences in maternal care. However, as expected, the age of the litter significantly affected two measures: time in nest and eating (p<0.05). The direction of the differences was consistent with dams spending more time in the nest with younger (P2) versus older (P6) pups. The F2 dams did not show any effects of DEHP dose lineage, but dams spent more time licking and grooming their litters on P2 than on P4 (p<0.05). Comparing maternal behaviors from dams in the three doses represented in both F0 and F2 we noted effects of generation in several measurements of maternal behavior. The effects of generation suggest a more active phenotype in the F0 dams. In sum, F0 dams spent less time in the nest (p<0.01), licking and grooming pups (p<0.01), and nursing (p<0.01) as compared with the F2 dams. F0 dams spent more time digging in the cage (p<0.01) than did the F2 dams. There were effects of the age of the litter for several measures. Dams spent more time on their nests (p<0.01), licking and grooming pups (p<0.01), and less time eating (p<0.001) when the pups were P2 as compared with P6 (Table 2, S1 Table).

**Table 2:** Maternal behavior in F0 and F2 dams.

	F1										F3					
		Control (10)		5 μg/kg (7)		40 μg/kg (9)		400 μg/kg (8)			Control (5)		5 μg/kg (5)		400 μg/kg (6)	
		*Mean*	*SEM*	*Mean*	*SEM*	*Mean*	*SEM*	*Mean*	*SEM*		*Mean*	*SEM*	*Mean*	*SEM*	*Mean*	*SEM*
**On Nest^**	P2*	0.47	0.13	0.44	0.14	0.44	0.10	0.51	0.11	**P2**	0.73	0.14	0.78	0.20	0.62	0.11
	P4*	0.33	0.12	0.37	0.17	0.41	0.14	0.33	0.08	**P4**	0.65	0.11	0.46	0.15	0.41	0.17
	P6*	0.26	0.09	0.31	0.12	0.21	0.12	0.13	0.05	**P6**	0.66	0.10	0.58	0.16	0.31	0.09
**Total nursing^**	**P2**	0.22	0.10	0.21	0.13	0.09	0.06	0.22	0.11	**P2**	0.34	0.12	0.53	0.22	0.19	0.09
	**P4**	0.13	0.09	0.19	0.15	0.12	0.08	0.07	0.05	**P4**	0.42	0.16	0.23	0.19	0.27	0.17
	**P6**	0.12	0.06	0.20	0.10	0.11	0.09	0.00	0.00	**P6**	0.47	0.12	0.29	0.16	0.09	0.06
**Licking**	**P2**	0.13	0.04	0.10	0.03	0.15	0.04	0.11	0.03	P2*	0.20	0.04	0.17	0.08	0.24	0.05
**and grooming^**	**P4**	0.09	0.04	0.06	0.03	0.16	0.06	0.06	0.02	P4*	0.12	0.02	0.15	0.08	0.04	0.02
	**P6**	0.05	0.03	0.06	0.01	0.06	0.03	0.04	0.02	P6*	0.11	0.01	0.15	0.07	0.11	0.03
**Digging**	**P2**	0.29	0.07	0.35	0.09	0.34	0.07	0.33	0.09	**P2**	0.14	0.09	0.11	0.10	0.13	0.04
	**P4**	0.32	0.07	0.43	0.13	0.35	0.10	0.41	0.07	**P4**	0.09	0.03	0.18	0.09	0.18	0.07
	**P6**	0.31	0.05	0.26	0.06	0.38	0.08	0.56	0.10	**P6**	0.11	0.04	0.20	0.09	0.32	0.09
**Eating**	P2*	0.20	0.06	0.15	0.05	0.17	0.03	0.10	0.03	**P2**	0.12	0.05	0.11	0.10	0.20	0.09
	P4*	0.29	0.06	0.18	0.06	0.20	0.07	0.23	0.04	**P4**	0.26	0.09	0.35	0.12	0.36	0.12
	P6*	0.40	0.08	0.34	0.06	0.37	0.08	0.28	0.07	**P6**	0.22	0.09	0.20	0.09	0.33	0.07

Mean proportion of maternal behaviors on each day measured: postnatal days 2 (P2), 4 (P4), and 6 (P6). Number of dams per group in ().

*Significant effect of litter age within generation, p<0.05.

^Significant effect of generation, p<0.01.

### Social interactions during pair-tests

#### F1 generation

**Fig 1:**
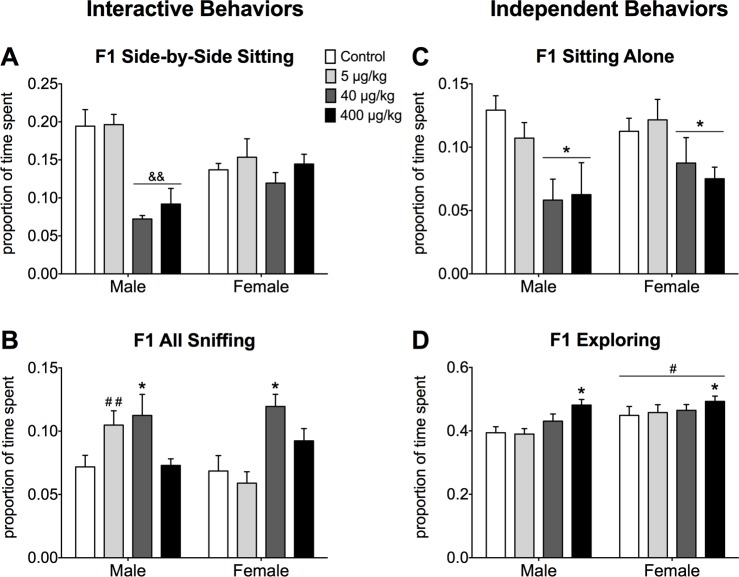
Effects of DEHP on juvenile pairs social behaviors tests in F1 mice. Mean ± SEM proportion of time spent A) sitting side-by-side, B) sniffing partner, C) sitting alone, and D) exploring the cage alone during a 30-min. test. White bars represent data from control mice, light grey bars show data from the lowest dose (5 μg/kg), dark grey bars show data from the medium dose (40 μg/kg), and black bars represent data from the highest dose (400 μg/kg). * Dose(s) significantly different from control and 5 μg/kg groups, p<0.05. && Significantly different from same-sex control and 5 μg/kg groups, p<0.05. #Significantly different from males, p<0.05. ##Significantly different from females of the same dose group, p<0.05. Numbers of F1 mice per group: Control male N = 18, Control female N = 14, 5 μg/kg Male N = 14, 5 μg/kg Female N = 14, 40 μg/kg Male N = 6, 40 μg/kg Female N = 12, 400 μg/kg Male N = 8, 400 μg/kg Female N = 6.

In general, F1 juvenile mice exposed in utero to the higher doses of DEHP (400 and 40 μg/kg) were less interactive than low dose (5 μg/kg) and control mice. We noted an effect of DEHP dose (p<0.001) on frequency of side-by-side sitting. Mice exposed to the medium and high doses exhibited less sitting side-by-side than mice in the low dose and control groups (Fig 1A). The effect of DEHP was limited to males of each dose group, creating a significant interaction between sex and dose (p<0.01). DEHP dose also affected the frequency of partner sniffing (p<0.001). F1 mice exposed to the medium dose sniffed their test partner more frequently than mice in the low dose and control groups (Fig 1B). Medium dose females had a higher sniffing frequency than low dose females and controls of both sexes, resulting in an interaction between sex and dose (p<0.05). Increased sniffing by low dose males led to a sex difference (males sniffing more than females) in mice exposed to the lowest dose. Finally, when we summed the frequencies of all interactive behaviors we noted an effect of sex; males were more interactive than females (p<0.01, S1 Table). However, there were no effects of dose, nor an interaction of dose and sex (p>0.05). The same statistical effects apply to the inverse measurement, summed independent behaviors.

In F1 mice, two independent behaviors were affected by DEHP dose. Control and low dose mice sat alone more frequently than either of the two higher DEHP dose groups (p<0.001, Fig 1C). For exploring the cage alone, we found effects of sex (p<0.05) and dose (p<0.05). Females explored more than males, and mice exposed to the highest dose of DEHP explored more frequently than both the control and low dose groups (Fig 1D).

#### F3 generation

**Fig 2:**
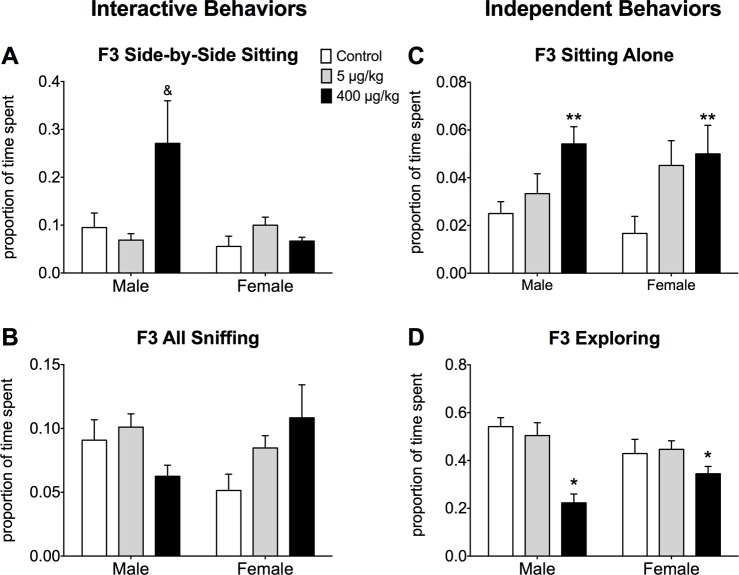
Effects of DEHP on juvenile pairs social behaviors tests in F3 mice. Mean ± SEM proportion of time spent A) sitting side-by-side, B) sniffing partner, C) sitting alone, and D) exploring the cage alone during a 30-min. test. White bars represent data from control mice; light grey bars show data from the lowest dose (5 μg/kg), and black bars represent data from the highest dose (400 μg/kg). *Significantly different from other groups, p<0.05. **Significantly different from same-sex controls, p<0.05. & Significantly different from all other groups except low DEHP dose females, p<0.05. Numbers of F3 mice per group: Control lineage male N = 10, Control lineage female N = 6, 5 μg/kg Lineage Male N = 8, 5 μg/kg Lineage Female N = 12, 400 μg/kg Lineage Male N = 4, 400 μg/kg Lineage Female N = 6.

Several behavioral differences were related to DEHP lineage in the F3 animals. In general, F1 and F3 effects of DEHP were reversed. In contrast to high dose (400 ug/kg) F1 groups, F3 males from the highest DEHP lineage in particular, were highly interactive. We found effects of sex (p<0.05), dose lineage (p<0.05), and an interaction (p<0.01) on frequencies of sitting together where males exhibited more side-by-side sitting than females and high dose lineage animals sat side-by-side with their partners more often than controls. The frequency of side-by-side sitting for the high dose male group was greater than all groups except low dose females (Fig 2A). There was no effect of ancestral DEHP exposure or sex on total sniffing frequencies (Fig 2B). However, the total frequency of all interactive behaviors was affected by sex (p<0.05), DEHP dose (p<0.001), and there was an interaction (p<0.05, S1 Table). Males interacted more frequently with their test partners than females, high dose animals displayed more interactive behaviors, and the high dose males drove both effects. The inverse was true for total independent behaviors: we observed fewer independent behaviors overall in high dose F3 males. For exploration, the most frequently displayed independent behavior; a dose lineage effect was revealed (p<0.001). F3 mice in the high dose lineage mice explored the cage alone less than control and low dose lineage groups (Fig 2D). For sitting alone, a less frequently displayed independent behavior, there was an effect of DEHP dose lineage (p<0.05). Mice in the high dose lineage sat alone more frequently than controls (Fig 2C).

#### Comparison of F1 and F3 behaviors

**Fig 3:**
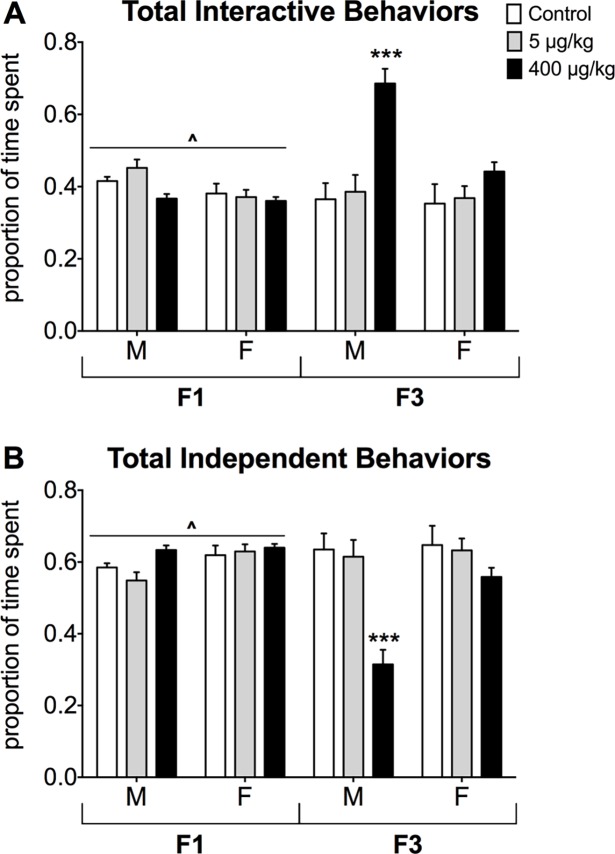
Comparison of total interactive and independent behaviors between generations. Mean ± SEM proportion of time spent engaging in A) all interactive behaviors (side-by-side sitting, social grooming, sniffing, following, approaching, crawling, and circling the partner). B) All independent behaviors (exploring the cage, self-grooming alone, sitting alone, and jumping). White bars represent data from control groups, light grey bars show data from the low dose lineage (5 μg/kg), and black bars represent data from the highest dose lineage (400 μg/kg). ^Significantly different from other generation, same group(s), p<0.05. ***Significantly different from all other groups (sexes and generations), p<0.05. M = Male, F = Female

To assess generational differences directly we compared F1 versus F3 mice in the three doses studied in both generations. Comparing F1 and F3 mice we found an effect of sex (p<0.01) and generation (p<0.001) for side-by-side sitting. Males were sat side-by-side more than females and F3 mice displayed this behavior more frequently than F1 mice. We also found interaction effects between dose and generation (p<0.001), sex and generation (p<0.05), and all three variables (p<0.001, S1 Table). Control and low dose mice in the F1 generation displayed more sitting side-by-side than F3 control and low dose mice. Additionally, F3 females displayed significantly less side-by-side sitting than F3 males and F1 mice of both sexes. For partner sniffing, there was a dose by sex interaction (p<0.01, S1 Table). Regardless of generation, control females sniffed their partners less often than low dose females and high dose males. For the total frequency of interactive behaviors, we found an effect of dose (p<0.001), a two-way interaction of dose and generation (p<0.001), and a three-way interaction of dose, sex, and generation (p<0.01, Fig 3A). Mice in the highest dose group were more interactive, particularly the F3 high dose males. Additionally, there were effects of sex (p<0.001) and generation (p<0.05) whereby males were more interactive than females, and F1 mice were less interactive than F3 mice. The statistical significance for total interactive behaviors is the same for the inverse measure, total independent behaviors (Fig 3B).

For independent behaviors, sitting alone was affected by generation (p<0.001): F1 mice sat alone significantly more often than F3 mice. Specifically, an interaction between generation and dose showed that control and low dose F1 mice sat alone more than mice in any other group (p<0.001, S1 Table). The data also revealed an effect of dose for exploring where high dose mice explored the cage less than control and low dose groups (p<0.01). An interaction between dose and generation (p<0.001, S1 Table) for exploring was caused by the high dose F3 mice, which explored less than any other groups. Additional statistics for social grooming, crawling, approach, circling, self-grooming alone, and jumping are listed in S2 Table.

#### Sex differences in elevated plus maze behavior

**Fig 4:**
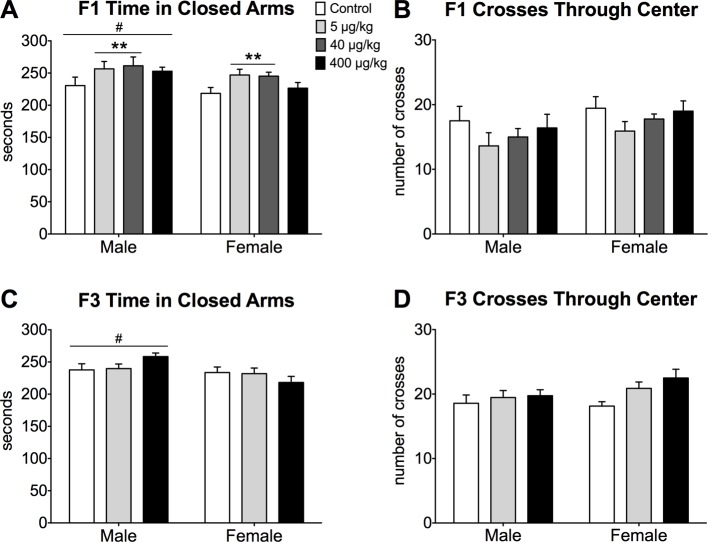
Elevated Plus Maze Behavior. Mean ± SEM for each group A) Time (sec) spent in the closed arm of the elevated plus maze (EPM) in F1 mice. B) Total number of crosses through the center of the EPM in F1 mice. C) Time (in seconds) spent in the closed arm of the EPM in F3 mice. D) Total number of crosses into the center of the EPM in F3 mice. White bars represent data from control mice, light grey bars show data from the lowest dose (5 μg/kg), dark grey bars show data from the medium dose (40 μg/kg), and black bars represent data from the highest dose (400 μg/kg). **Doses significantly different from controls, p<0.05. #Significantly different from other sex, p<0.05. Numbers of F1 mice per group: Control male N = 8, Control female N = 9, 5 μg/kg Male N = 8, 5 μg/kg Female N = 11, 40 μg/kg Male N = 5, 40 μg/kg Female N = 9, 400 μg/kg Male N = 5, 400 μg/kg Female N = 4. Numbers of F3 mice per group: Control male N = 7, Control female N = 7, 5 μg/kg Lineage Male N = 13, 5 μg/kg Lineage Female N = 9, 400 μg/kg Lineage Male N = 8, 400 μg/kg Lineage Female N = 12.

We found a sex difference on the EPM in juvenile mice. F1 juvenile males spent more time in the closed arms than females, resulting in an effect of sex (p<0.05). DEHP dose affected the amount of time spent in the closed arms (p<0.05) where low and medium dose groups spent more time in the closed arms than controls (Fig 4A). There was a trend for an effect of sex in the number of crosses through the center of the maze, an indirect measure of activity (p = 0.06), where females tended to be more active than males on the maze. There were no effects of DEHP dose, nor was there an interaction of sex and dose on crosses through the center (p>0.05, Fig 4B). There were no significant effects of DEHP on time spent in the open arms or in the center portion of the maze (p>0.05, S1 Table). In F3 mice, we also observed that males spent more time in the closed arms than females (p<0.05, Fig 4C). There was no significant dose effect, nor was there an interaction between dose and sex (p>0.05). Center crosses in F3 mice were not affected by sex, dose, nor was an interaction present (p>0.05; Fig 4D). DEHP lineage did not affect time spent in the open arms or center of the maze in the F3 generation (p>0.05, S1 Table).

A between-generation comparison of the doses common to all groups yielded similar statistical results. Males spent more time in the closed arms than females (p<0.01; Fig 4A and 4C). There was no effect of generation or dose (p>0.05) for closed time. Sex and generation both affected the number of crosses through the center of the maze (p<0.05, S1 Table). Overall, females crossed through the center of the maze more than males (p<0.05) and F3 mice made more crosses through the center than F1 mice (p<0.05, Fig 4B and 4D). Across F1 and F3 generations, females spent more time in the center of the maze than males (p<0.01), but there was no significant sex difference in time spent in the open arms (p>0.05). Likewise, there were no effects of dose or generation for either measure (p>0.05, S1 Table).

## Discussion

Here we report that neonatal exposure to DEHP has dose-, sex-, and generation-dependent effects on social behavior, anxiety-like behavior, and AGI in mice. While some of the measures we examined have been reported on previously, here, we used very low doses, on the order of 500-fold lower than used in other animal studies [12, 34, 35]. Daily oral DEHP exposure to pregnant females resulted in serum metabolite levels within the range of human exposure. Serum MEHP levels in embryos from the highest dose averaged 96.7 ± 36.9 ng/mL, much lower than levels in human cord blood serum which at birth average 0.52 ± 0.61 μg/mL [41]. We found no significant effect of DEHP exposure on maternal behavior in the F1 or F3 generations, indicating that the transgenerational effects of DEHP are likely not socially transmitted via differences in maternal care of offspring. However, we recognize that maternal behavior was only measured during the first week, and that future studies could evaluate this effect throughout the entire lactation period.

Our social behavior results are consistent with epidemiological studies in human populations indicating significant impacts of early life exposure to phthalates on neurobehavioral outcomes [20–26]. Several of the individual behavioral measures we observed were modified by gestational and lactational exposure to DEHP, and some behavioral differences persisted to the F3 generation. F1 generation mice exposed to the two highest doses (40 and 400 μg/kg) displayed less frequent side-by-side sitting and sitting alone than control animals. High dose animals also explored the cage more than controls, which could indicate a difference in overall activity. However, our indirect activity data from the EPM do not support this interpretation. Additionally, we noted an inverted “U”-shaped response for males in the frequency of sniffing their partners, which is suggestive of a non-monotonic dose response.

The only existing data comparable to ours are from research conducted in ICR mice that received doses of DEHP given by gavage during puberty (P28-P42). In this study, males exposed to 50 mg/kg DEHP engaged in more “social play” and spent more time in social investigation time than controls [35]. Females in the same study exposed to several doses of DEHP (1, 10, 50 or 200 mg/kg) displayed less investigation than controls [33, 35]. In contrast, in this study social sniffing frequency was increased by the medium dose (40 ug/kg, a greater than 1000-fold lower dose) in both sexes. It is difficult to directly compare the results of these studies as the age of subjects, time of exposure, doses, route of administration, and mouse strain are different. Exposure to DEHP during puberty cannot affect early neurodevelopmental processes occurring during the critical period of gestation, but it may affect the maturation of reproductive system and levels of circulating sex steroids, which could alter social behaviors [42]. Additionally, there are documented strain-dependent differences in social interaction behaviors as well as sensitivity to EDCs, including DEHP [43–45]. Despite these differences, both studies demonstrate significant effects of DEHP exposure during critical periods on social behaviors in mice.

We found transgenerational effects of DEHP on several specific behaviors; side-by-side sitting, sitting alone, and exploring. The mice in the high dose lineage group were primarily responsible for these effects. High dose lineage mice of both sexes sat alone more frequently and explored less during the test compared to controls, whereas side-by-side sitting was increased in high dose lineage males only. When all measurements for interactive and independent behaviors were combined, we found a transgenerational enhancement in social interactions and a reduction in independent behaviors in the high dose lineage males. The only other study on transgenerational behaviors in DEHP lineages is from our group [36]. In that study, a higher (150 mg/kg) dose was given by gavage to F0 dams and the exposure was restricted to gestational days 7–14. Juvenile F3 DEHP lineage males displayed more digging and less self-grooming than controls in a similar social interaction test.

Perhaps the most interesting aspect of our data is the shift in behavioral patterns between F1 and F3 offspring. In general, in the highest dose group, F1 mice were less interactive and more independent while F3 mice had the reversed pattern. It is not unusual for transgenerational effects of EDCs to be enhanced or even reversed over the generations [46–48]. For example, in our studies on the transgenerational actions of bisphenol A (BPA) we noted a behavioral shift between generations exposed to BPA versus control diets in social interactions, using a similar behavioral testing paradigm [48]. Like F1 DEHP mice, in F1 juveniles exposed to BPA during gestation we note fewer interactive and more independent behaviors than controls. However, over the generations we found a shift. In the F2 and F4 generations, the BPA lineage mice were more interactive and less independent than controls. We have since reported a transgenerational effect of BPA on social recognition. Juvenile mice exposed during gestation to BPA exhibit delayed habituation to the familiar stimulus animal in a social recognition task. F3 offspring show this same delay, but in addition, they failed to recognize the novel stimulus animal in the last trial. Additionally, F3 BPA lineage mice were more active in the open field test than controls, but there were no significant effects on activity in the F1 generation [49]. Unlike the BPA data set, transgenerational actions of DEHP were largely restricted to males. These data illustrate that EDC actions on the developing brain (in F1) versus long-term effects on germ cells (F3 and beyond) can result in different behavioral phenotypes.

Transgenerational effects of low doses of EDCs are mainly attributed to epigenetic changes, presumably at the level of the germ cell. Exposure to DEHP in utero produced increased global DNA methylation and increased expression of DNA methyltransferases (DNMTs) in fetal testes [50]. In a different study DEHP exposure during gestation increased DNMT expression in adult testes of F1 and F2 rat offspring [51]. Additionally, gestational exposure to 40 μg/kg DEHP (our medium dose) in mice resulted in heritable changes in DNA methylation of imprinted genes in primordial germ cells [52]. Prenatal phthalate exposure is also associated with altered DNA methylation patterns in human placenta [53–55] and cord blood samples [56]. Studies of other EDCs have characterized multigenerational and transgenerational mechanisms [57–61], but in general more research on different aspects of epigenetic regulation (i.e. miRNA, histone modifications, long non-coding RNAs, etc.) is necessary to determine how changes in behavior are inherited over generations.

DEHP has documented anti-androgenic actions. In humans, maternal phthalate metabolites in the first trimester of pregnancy are negatively correlated with AGD in infant boys [17, 19] and with human chorionic gonadotropins, which in turn correlated with shorter AGD in boys and longer AGD in girls [62]. In this study, we observed effects on AGI in both sexes. Based on dose-response studies conducted in rats and mice [14, 16], we hypothesized that we would observe a non-monotonic dose-response for AGI: the higher doses (40 and 400 μg/kg) would exert anti-androgenic actions whereas the lower dose (5 μg/kg) would have an androgenic effect. However, both low and high doses of DEHP had an anti-androgenic effect in F1 generation males and females. The AGIs in low dose males and high dose females were significantly shorter than in the same-sex controls. These findings suggest sex differences in the sensitivity of the developing anogenital region to DEHP in utero. In a similar study in CD-1 mice, AGI was increased in male fetuses exposed to 5 μg/kg DEHP from gestational days 9–18, while a larger dose had no effect [14]. Several differences in experimental design may explain the discrepancy between this result and ours. In addition to using a different strain of mouse and a shorter exposure window, only male fetuses adjacent to one other male fetus (1M) in the uterus were measured, whereas we could not account for variability due to intrauterine position. For females of the F3 generation, ancestral exposure to low doses of DEHP was associated with increased AGI and decreased body weight compared with control females. It is unclear whether the dose effect on AGI in low dose F3 females is a secondary effect of decreased body weight or a genuine effect of dose lineage on reproductive development. In a previous study, higher doses of DEHP (150 or 200 mg/kg) were given during a more limited exposure period (gestational days 7–14). Transgenerationally, F3 males from the 150 mg/kg lineage had significantly larger AGI and F3 200 mg/kg lineage males had increased body weights, whereas neither measure was significantly affected by DEHP lineage in F3 females [36].

Lastly, we recorded an increase in anxiety-like behavior in both sexes of the low and medium doses specifically in the F1 generation. This is the first example of DEHP affecting anxiety-like behavior at doses this low. To date, there are several reports of increased anxiety-like behavior in mice directly exposed to higher doses of DEHP (10–540 mg/kg) [12, 34, 63, 64]. The mechanism underlying the enhanced anxiety-like behavior is not clear. One way DEHP may influence anxiety-like behavior is by disturbing the hypothalamic-pituitary-adrenal (HPA) axis. MEHP regulates glucocorticoid metabolism by inhibiting 11 beta-hydroxysteroid dehydrogenase type 2 (11β-HSD2), the enzyme responsible for inactivating the stress hormone corticosterone [65, 66]. 11β-HSD2 is highly expressed in the placenta and embryonic brain and serves as a barrier to protect developing tissues from excess maternal corticosterone [67]. Rats exposed to high levels of corticosterone during gestation and early life spend more time in the closed arms of the elevated plus maze as adults [68]. Dexamethasone, a synthetic glucocorticoid, amplifies the effect of prenatal phthalate exposure on reproductive outcomes (decreased testosterone production, shortened AGD, etc.) in mice [69]. Similarly, maternal stress levels and DEHP exposure during pregnancy can interact in complex ways to affect AGD in human male infants [70, 71]. These data support the hypothesis that excess gestational corticosterone may potentiate the effects of DEHP on anxiety-like behavior in the elevated plus maze. We have previously reported that ancestral exposure to a high dose of DEHP (150 mg/kg/day) is associated with decreased serum corticosterone at baseline and following restraint stress in DEHP lineage females as compared with controls, despite observing no significant anxiety-like phenotype on the elevated plus maze [36].

Some of these changes in behavior may be explained by the impact of DEHP and its metabolites on neurodevelopment, of which there are several examples. Dopaminergic neuron numbers and tyrosine hydroxylase (TH) immunoreactivity in the midbrain is decreased in 6-week-old mice following 1 mg/kg daily gestational and neonatal administration of DEHP [72]. Furthermore, pubertal exposure to DEHP at doses ranging from 1–200 mg/kg decreased dopamine receptor D2 protein in the striatum of adult female mice, accompanied by significant alterations of social and anxiety-like behavior [33]. Additionally, mouse neurons co-cultured with astrocytes have increased oxidative stress markers and increased gliosis following exposure to biologically relevant DEHP levels [73]. DEHP affects the brain in widespread ways that may have extensive effects on neurodevelopment and behavior, but the mechanisms for effects must be more thoroughly explored.

### Conclusions

This dose-response response study identified transgenerational effects of perinatal DEHP exposure on social behaviors in juvenile mice. We also showed increased anxiety-like behavior and decreased AGI in mice directly exposed to DEHP. This is the first study to report effects of DEHP exposure on social behavior in F1 and F3 generations at doses this low. Now that behavioral changes in each dose, sex, and generation have been identified, future studies will focus more directly on mRNA and protein changes in the brain in response to specific doses within this exposure model. This will provide insight into the impact of DEHP on humans, especially during critical periods of development.

## Supporting information

S1 Table Supplemental F Statistics. F statistics, degrees of freedom, and symbolic representations of p values for each factor analyzed in the results section. * p<0.05; ** p<0.01; *** p<0.001; ^ p = 0.06
(XLSX)

S2 Table Statistical values for additional social behavior measures. F statistics, degrees of freedom, and p values for each effect for the analyses of F1, F3, and F1 vs. F3 for social grooming, crawl, approach, circling, self-grooming alone, and jumping.
(XLSX)
